# My Dear Doctor

**Published:** 1901-01

**Authors:** 


My Dear Doctor.—Didn't you get a little bill in October—which you have pigeon-holed or thrown away? Its a *heap* of labor to have to make out bills,—please remit.

---

To the Doctors: Mrs. S. Terry, so well known to the profession, and so popular,—who *always* attends the meetings of the State and other medical societies—is now representing, traveling for and soliciting subscriptions for the *Red Back.* We commend her to the profession everywhere, and will appreciate any and all attentions and courtesies shown her, and all subscriptions given her.

---

We want a few copies of the *Red Back* for August, September, October, November and December, 1900. The demand has exceeded the supply ever since July, and we are printing three hundred copies a month additional, this year. For every copy sent us we will credit the sender with one month on his subscription account, and will pray for him when he gets the *grippe,* or the grippe gets him and he lets go *his* grip—and goes to look after his patients who have "gone before."

